# Participation but not success in youth and junior World Championships is important for overall ranking in the biathlon World Cup during adult age

**DOI:** 10.3389/fspor.2024.1507146

**Published:** 2024-11-27

**Authors:** Malin Jonsson Kårström

**Affiliations:** Swedish Winter Sports Research Centre, Department of Health Sciences, Mid Sweden University, Östersund, Sweden

**Keywords:** adolescence, cross-country skiing, early specialization, elite athletes, predicting success

## Abstract

**Purpose:**

The aim of this study was to investigate if competition rank in the youth (16–19 years) and junior (20–22 years) World Championships (WCH) and age of the athlete when reaching certain World Cup (WC) performance milestones (debut and top 40, 15, 6 and 3) were related to future World Cup total (WCT) performance.

**Methods:**

All data was obtained from the International Biathlon Unions database. The biathletes ranked top 30 in the WCT (82 men, 98 women) during 10 consecutive competition seasons were selected for further analysis. Biathletes were divided into performance groups due to their best WCT ranking (WCT6, rank 1–6; WCT15, rank 7–15; WCT30, rank 16–30). The relation between youth and junior WCH ranking and age when reaching the WC performance milestones with WCT performance was investigated.

**Results:**

63.3% and 86.1% of the biathletes in the WCT top 30 competed in the youth and/or junior WCH, respectively, but the correlation between junior and senior ranking was low. WCT6 reached most of the WC performance milestones at a younger age compared to WCT15 and WCT30 (*p* > 0.05) and reaching WC top15 at a younger age increased the chance of reaching WCT6 in the future.

**Conclusion:**

It seems beneficial to compete internationally (i.e., participation in youth/junior WCH) and reach certain WC performance milestones at a young age to achieve a high ranking in the WCT during adult age. This highlights that biathletes need to have a certain performance level during junior years, although ranking in youth/junior WCH is not a prediction for senior WCT success.

## Introduction

Biathlon combines two completely different tasks: endurance-based cross-country (XC) skiing and the fine motor control of rifle marksmanship. During a biathlon competition, the athlete skis 6–20 km (depending on competition format and sex) in the skating technique while carrying the rifle on their back (minimum weight 3.5 kg). The skiing is separated by 2 or 4 shooting bouts consisting of 5 shots each and alternates between the prone and standing shooting position. For each missed target, the biathlete has to ski a penalty loop of 75 or 150 m depending on the competition format) or receive a time penalty of usually 1 min. The overall performance in biathlon depends on three factors: skiing speed, shooting speed, and shooting accuracy (1).

The highest competition league in biathlon is the World Cup (WC), and a high ranking in the World Cup total (WCT) is an important goal for many biathletes, together with the World Championships (WCH) and the Olympic Games. The second highest league is the International Biathlon Union Cup (IBUC), and the biathletes qualify with their results from the IBUC for the limited WC quotas (2–6 depending on country and sex) (2). An athlete ranked 20–25 in the IBUC is usually placed around 60–70 in the WC and there is a significant correlation between the ranking in the two different competition leagues (*r* = 0.68–0.75) (3). In biathlon, athletes at the age of 22 years and younger are divided into two competition classes, youth (16–19 years) and junior (20–22 years), when competing at an international level (2). The WCH for youth and juniors is arranged yearly, consisting of both individual competitions and relays. The biathletes start to compete in the senior class at the age of 23 years, but it is possible to compete in the IBUC and WC also as juniors if the athlete reaches the qualification criteria for the different types of competitions (2).

Only two case studies have been published regarding performance and training development in World-class biathletes over a longer period of time (male biathlete 21–31 years of age; female biathlete 17–33 years of age) (4, 5). The female biathlete started with biathlon early (age 10) and competed in the WCH two times as a junior, winning one medal. She made her WC debut at the age of 21 years and had her peak performance between the ages of 31–33 years (5). The male biathlete competed two times in the youth and junior WCH (one time in each class), made his WC debut at 19 years old, and had his peak performance during the ages 24–30 years old (4, 6). These two studies indicate that ranking at the youth and junior WHC may not be important for future success in biathlon and that the age of the biathletes at peak performance may be relatively high.

Early specialization is a well-debated area, and the link between junior and senior performance is still unclear and may differ between different types of sports. Barth et al. (7) demonstrated in a meta-analysis including different types of sports (e.g., basketball, soccer, athletics, rowing, swimming) that junior (up to 17–19 years of age, depending on sport) performance only explained 2.2% of the reliable variance in senior performance and that the correlation between junior and senior performance was even lower for the younger junior age categories. On the contrary, Li et al. (8) showed that more than 60% of the junior medalists (up to 18 years of age) in international competitions and 90% of the early achievers (competing in senior competitions as a junior) in combat sports also won international medals at senior age.

The link between junior and senior performance has been investigated in several different sports, but with the greatest emphasis on team sports (7, 9). However, no previous study has investigated the relationship between junior and senior performance in biathlon. The primary aim of this study was therefore, to investigate if competition rank in the youth and junior WCH was related to senior biathlon performance in the WCT. A second aim was to investigate if the age of the athlete when reaching certain performance milestones in IBUC (debut) and WC (debut, top 40, 15, 6 and 3) was related to WCT performance later in the biathlon career.

## Methods

All data were obtained from the datacenter of the International Biathlon Union (IBU) https://www.biathlonresults.com/#/start which is an openly available public domain. Permission to use the data for scientific purposes was granted by the IBU. The biathletes placed 1–30 in the WCT during 10 consecutive seasons from 2013/2014 to 2022/2023 were selected for further analysis. Many biathletes reached a ranking in the top 30 during several competition seasons, giving a total of 180 biathletes (82 men and 98 women) ranked top 30 in the WCT during these 10 competition seasons. Date of birth and the age of the athlete when starting with biathlon (self-reported by the athlete), results from youth and junior WCH, and results from IBUC and WC competitions were obtained from the IBU data center. The biathletes were divided into three performance groups based on their best ranking in the WCT during the selected competition seasons: rank 1–6 (WCT6), rank 7–15 (WCT15), and rank 16–30 (WCT30). Based on the biathlete's best individual ranking in each WCH competition class (youth and junior) they were assigned to a junior performance group: *participation* (best ranking lower than 15), *top 15*, *top 6*, and *top 3*. For individual IBUC and WC competitions, the age of the biathlete was calculated for when a certain milestone was reached for the first time; *IBUC and WC debut*, and the following WC results: *first time placed top 40* (first WC points), *top 15*, *top 6* (first flower ceremony) and *top 3* (first podium).

### Data and statistical analysis

All statistical analyses were processed using IBM SPSS Statistics (version 29.0, IBM Corporation, NY, USA). All variables were tested for normality using Shapiro-Wilks test, showing a normal distribution for all variables. To investigate if ranking at the youth and junior WCH or the age of the athletes at the defined milestones differed among the performance groups (WCT6, WCT15 and WCT30), a one-way between subjects ANOVA was used. In the case of a significant result of the ANOVA, a Bonferroni post-hoc test was used to identify specific differences between groups. Effect sizes were calculated using partial Eta square (ηp^2^) with the criteria 0.01 = small effect, 0.06 = moderate effect, and 0.138 = large effect (10). In addition, a multi-nominal regression analysis was used to investigate the association between ranking at youth and junior WCH and athletes' age for IBUC and WC milestones to the group rank of WCT performance (WCT6, WCT15, and WCT30). The analysis was performed using separate models for youth/junior WCH performance and IBUC/WC milestones. The reference group, i.e., the base group that the other groups were compared to, was set to WCT6 for all analyses. Pearson’s correlation coefficients (*r*) was used to investigate the relationship between the best ranking in the youth and junior WCH with the best ranking in the WCT, with the strength of the correlations interpreted as small (*r* = 0.10–0.29), medium (*r* = 0.30–0.49) and large (*r* = 0.50–1.00) (10). The ANOVA and regression analysis were performed with men and women separated and as a whole group. Ranking from youth and junior WCH and age at IBUC and WC milestones were compared between sexes using an independent samples *t*-test. Data are presented as mean ± standard deviation (SD) or odds ratio (OR) with confidence intervals (95%, CI). The level of significance was set at *α* ≤ 0.05.

## Results

### Performance groups

49 biathletes (24 men and 25 women) were ranked 1–6 (group WCT6) in the WCT at least one time during the seasons 2013/2014 until 2022/2023, while 48 (25 men and 23 women) and 83 (33 men and 50 women) athletes had the highest ranking of 7–15 (group WCT15) and 16–30 (group WCT30), respectively. The 180 biathletes represented 23 different nations, with the largest number of athletes from Germany (24 athletes), Russia (23 athletes), Norway (22 athletes) and France (20 athletes).

### Youth and junior World Championships

**Figure 1:**
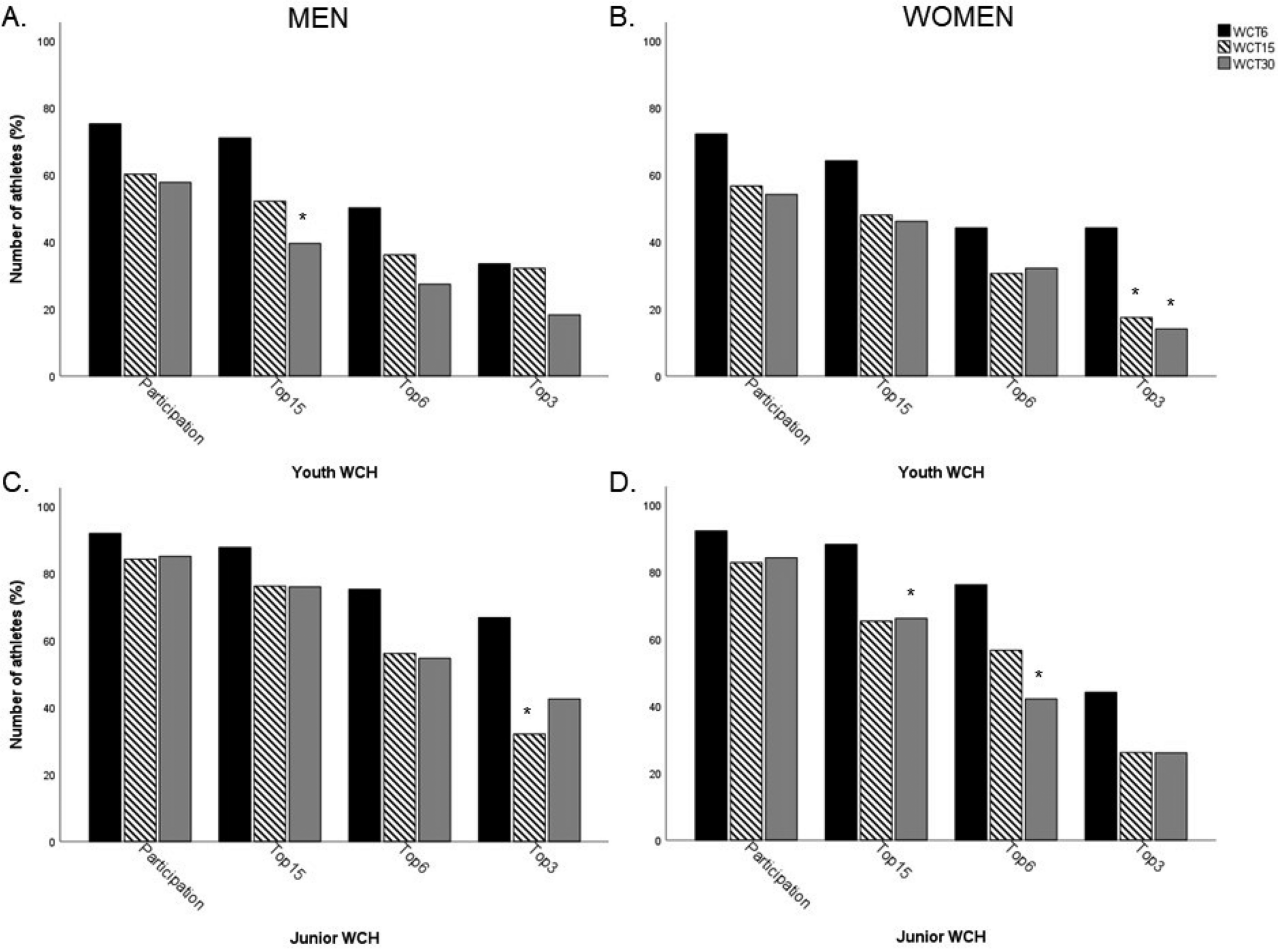
Amount of the total number of biathletes (82 men and 98 women) in the three different performance groups [World Cup total top 6 (WCT6), ranking 7–15 (WCT15) and ranking 16–30 (WCT30)] that participated in the youth (**A**—men, **B**—women) and junior (**C**—men, **D**—women) World Championships (WCH) and their best individual performance (reaching top 15, 6 and 3) during the two WCH types. * = significantly different to WCT6, *p* < 0.05.

**Figure 2:**
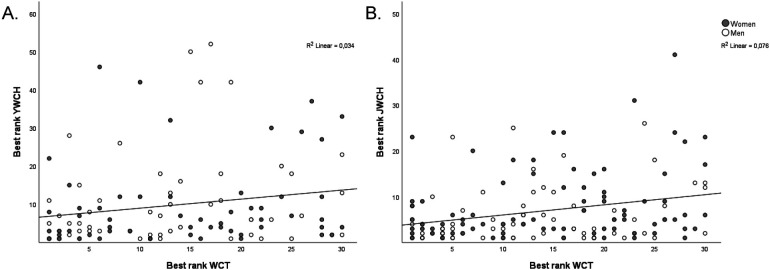
The relationship between the best ranking in youth **(A)** and junior **(B)** World Championships (YWCH and JWCH) with the highest ranking in the World Cup total (WCT). Grey markers symbolize women, while black circles symbolize men.

114 (63.3%) and 155 (86.1%) of the biathletes ranked top 30 in the WCT competed in the youth and junior WCH, respectively, with no differences between the WCT performance groups (Figure 1). This means that 66 (36.7%) and 25 (13.9%) biathletes ranked top 30 in the WCT did not compete in the youth and junior WCH. 44 of the athletes (24.4%) won at least one medal at the youth WCH, winning a total of 84 medals. In the junior WCH, 68 of the athletes in this study (37.8%) won at least one medal, winning a total of 145 medals. The performance group for the WCT was not associated with the best individual ranking in youth or junior WCH (Table 1). The relationship between the best ranking in youth and junior WCH with the highest ranking in WCT was *r* = 0.18 (*p* = 0.049) and *r* = 0.28 (*p* < 0.001), respectively (Figure 2). Men achieved a better top ranking in the junior WCH compared to the women (5.6 vs. 7.9, *p* = 0.042), while there was no difference between sexes in youth WCH (*p* = 0.712).

**Table 1:** Multinominal logistic regression for the athletes age when starting with biathlon (BIA) and the best individual rank in the youth (YWCH) and junior World Championships (JWCH) in relation to performance group [according to best ranking in the World Cup total (WCT6, WCT15 and WCT30)]. Data are reported as odds ratio (OR) with a 95% confidence interval (95% CI) and *p*-values.

Group	Women			Men		
	OR	95% CI	*p*-value	OR	95% CI	*p*-values
WCT15-WCT6						
Age Start BIA	1.14	0.84–1.56	0.391	1.05	0.81–1.36	0.732
Best rank YWCH	0.99	0.91–1.07	0.814	1.04	0.95–1.13	0.423
Best rank JWCH	1.19	0.95–1.49	0.130	1.01	0.87–1.18	0.905
WCT30-WCT6						
Age Start BIA	1.21	0.92–1.59	0.171	1.04	0.80–1.34	0.789
Best rank YWCH	0.97	0.90–1.05	0.473	1.05	0.97–1.14	0.259
Best rank JWCH	1.24	0.99–1.54	0.058	1.04	0.91–1.20	0.546

### IBUC and WC milestones

**Figure 3:**
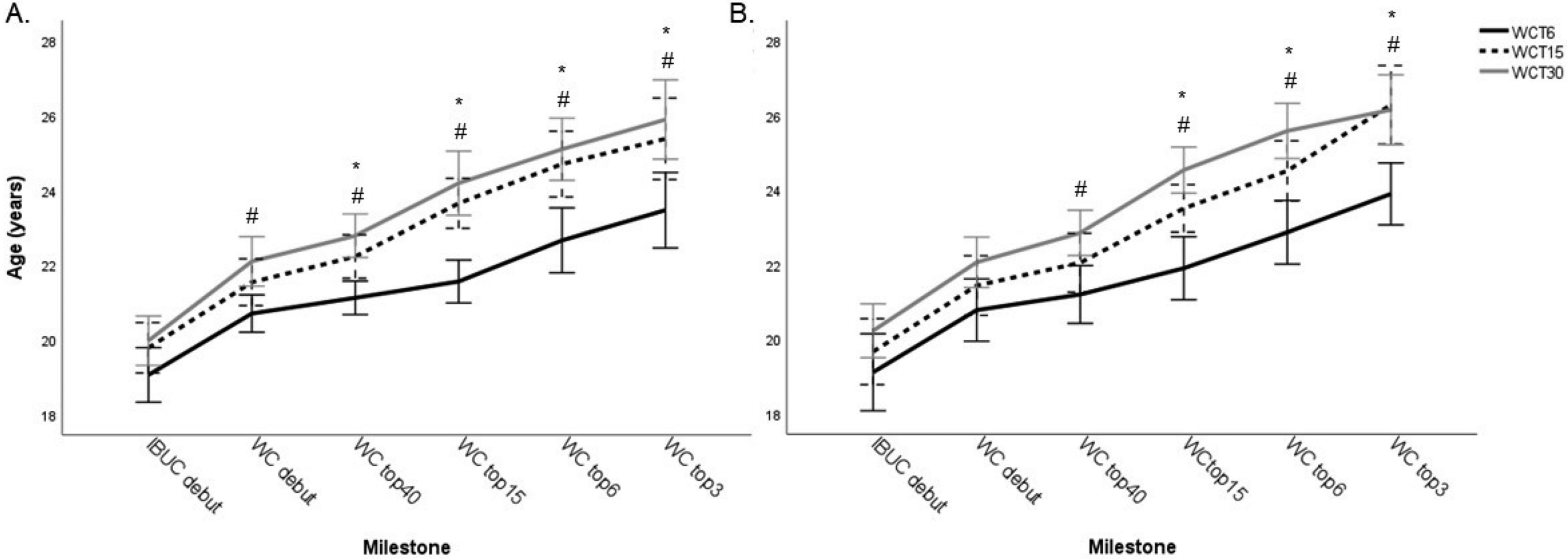
The mean age ± standard deviation of the athletes (**A**—men, **B**—women) when reaching the different competition milestones for the first time divided into the different performance groups according to best world Cup total (WCT) ranking, WCT6 (black solid line), WCT15 (black dotted line) and WCT30 (grey solid line). IBUC, International Biathlon Union Cup; WC, World Cup, * = significant difference between WCT6 and WCT15, p < 0.05, ^#^ = significant difference between WCT6 and WCT30, p < 0.05.

For the group in total, WCT6 made their debut in the IBUC at a younger age compared to WCT30 (Table 2). However, there was no difference between WCT performance groups in age at the IBUC debut when analyzing the sexes separately (Figure 3). For the group in total, WCT6 reached most of the WC milestones at a lower age compared to WCT15 and WCT30 (Table 2). The same pattern was shown when analyzing the sexes separately, with WCT6 reaching top 15, 6, and 3 at a younger age for both men and women, compared to WCT15 and WCT30 (Figure 3). For both men and women, reaching WCT6 was associated with the athlete's age when reaching top 15 for the first time in WC, compared to WCT15 and WCT30 (Table 3). No other significant association between performance milestones and the WCT performance group was found (Table 3). No difference between sexes was found for age at any of the IBUC or WC milestone achievements (*p* = 0.213–0.800).

**Table 2:** The mean age ± standard deviation of the biathletes when reaching different competition milestones for the first time. The biathletes are divided into performance groups according to their best rank in the World Cup Total (WCT); WCT6, WCT15 and WCT15.

	Performance group				
	WCT6	WCT15	WCT30	*p*-value	Effect size
Age start biathlon (years)	12.8 ± 3.8	14.9 ± 3.8^a^	14.2 ± 4.1	0.038	0.038
Age IBUC debut (years)	19.1 ± 2.0	19.7 ± 1.8	20.1 ± 2.2^a^	0.031	0.042
Age WC debut (years)	20.7 ± 1.7	21.5 ± 1.7	22.1 ± 2.2^a^	<0.001	0.077
Age WC top 40 (years)	21.2 ± 1.6	22.1 ± 1.7^a^	22.8 ± 1.9^a^	<0.001	0.134
Age WC top15 (years)	21.7 ± 1.8	23.6 ± 1.6^a^	24.4 ± 2.3^a^	<0.001	0.242
Age WC top 6 (years)	22.8 ± 2.1	24.6 ± 2.0^a^	25.4 ± 2.3^a^	<0.001	0.208
Age WC top 3 (years)	23.7 ± 2.2	25.8 ± 2.5^a^	26.0 ± 2.5^a^	<0.001	0.168

^a^ Significantly different to WCT6, *p* < 0.05.

**Table 3:** Multinominal logistic regression for the athletes age when reaching the different competition milestones for the first time in relation to performance group [according to best ranking in the World Cup total (WCT6, WCT15 and WCT30)]. Data are reported as odds ratio (OR) with a 95% confidence interval (95% CI) and *p*-values.

Group	Women			Men		
	OR	95% CI	*p*-value	OR	95% CI	*p*-values
WCT15-WCT6						
Age IBUC debut	1.09	0.66–1.80	0.728	0.96	0.50–1.82	0.888
Age WC debut	0.86	0.32–2.30	0.761	1.30	0.34–5.02	0.705
Age WC top 40	0.60	0.20–1.78	0.356	0.31	0.06–1.73	0.183
Age WC top 15	2.44	1.00–5.98	0.050	5.39	1.69–17.21	0.004
Age WC top 6	0.84	0.45–1.56	0.572	0.93	0.44–1.98	0.854
Age WC top 3	1.50	0.94–2.40	0.090	0.89	0.52–1.55	0.687
WCT30-WCT6						
Age IBUC debut	1.18	0.73–1.93	0.501	0.97	0.51–1.87	0.933
Age WC debut	0.60	0.23–1.60	0.308	2.31	0.60–8.88	0.222
Age WC top 40	0.93	0.31–2.79	0.903	0.46	0.07–2.92	0.407
Age WC top 15	2.83	1.15–7.00	0.024	2.15	0.69–6.71	0.189
Age WC top 6	0.96	0.51–1.92	0.964	1.05	0.54–2.02	0.889
Age WC top 3	1.08	0.65–1.78	0.768	1.24	0.71–2.15	0.453

IBUC, International Biathlon Union Cup; WC, World Cup.

## Discussion

The aim of this study was to investigate if competition rank in the youth and junior WCH and the age of the athlete when reaching certain IBUC and WC performance milestones were related to WCT performance later in the biathlon career. The main results of the study were as follows: (1) out of the 180 biathletes analyzed in this study, 63% competed in the youth WHC while 86% of the biathletes competed in the junior WCH, (2) the ranking in youth and junior WCH was not different between the WCT performance groups; (3) the best performance group, WCT6, reached a majority of the WC performance milestones (top 40, 15, 6, and 3) at a younger age compared to WCT15 and WCT30; and (4) a younger age when reaching the top15 in WC for the first time was associated with an increased chance to reach the best performance group (WCT6) in the future.

In this study, the results showed that youth and junior WCH ranking was not a predictor for future performance in the WCT and that the correlation between the best ranking in the youth and junior WCH and WCT ranking was significant but low (*r* = 0.18 and 0.28, respectively). This is in line with a previous meta-analysis, demonstrating that junior performance explained only 2.2% of the reliable variance in senior performance and that the correlation between junior and senior performance was lower with younger junior athletes (7). Previous research has shown that in many sports, successful juniors and successful seniors are, to the largest part, two disparate populations (11). Only 17%–34% of the best-performing juniors (or pre-juniors aged 14–16 years) demonstrated a successful transition (i.e., reached the same level as seniors) in swimming (12–16), soccer, volleyball and judo (17). Nevertheless, previous research in swimming has also shown that out of the few athletes that had a successful transition, the swimmers that were performing well as juniors had a slightly better chance of becoming successful senior athletes (13, 16).

Most of the athletes ranked top 30 in the WCT hade been competing in the youth and/or junior WCH previously. Previous research in swimming has shown that athletes who had previously participated in the junior WCH showed significantly better performance times in swimming as seniors compared to athletes who did not qualify for the junior WCH (18), which is in line with the result of the previous study. Born et al. (19) have discussed in their article (also from a swimming context), that since there is a limitation in the annual progression in performance, the athlete needs to have a certain performance level as a junior, to have the chance to reach the highest level as a senior. This is also supported by the present study where the biathletes probably need to reach a certain performance level at junior age (i.e., qualifying for the youth/junior WCH) to increase the chance of reaching a high ranking in the WCT as seniors, but that the ranking in the youth/junior WCH itself is not a predictor for future success.

The athletes in the present study started with biathlon at an average age of 13–15 years (depending on performance group), but no available data showed when the athletes specialized in biathlon. Previous research has shown that successful junior athletes started playing their main sport at an earlier age, engaged more in main-sport practice and less in other-sport practice, and demonstrated faster initial progress compared to their lower-performing peers (9, 20, 21). The opposite pattern has been shown in successful seniors, where athletes normally had more childhood/adolescent multi-sport involvement, a later main-sport start, less main-sport practice and slower initial progress (9, 21–23). This has also been the case for one of the most successful female biathlete, who had an active childhood with a variety of sport activities and a late main-sport specialization (18 years old) (5). In swimming, previous research has shown that successful senior athletes demonstrated continuous improvement in their performance at older ages, compared to their less successful counterparts (24). This seems to be the case also in biathlon, since the successful biathletes in this study reached the different performance milestones gradually, with the mean age being ∼24–26 years when reaching the podium for the first time, depending on the WCT performance group.

The result of this study demonstrates that the age of the athletes when reaching certain WC performance milestones seems to be of importance for future performance in the WCT. The results demonstrated a difference in age between WCT6 and WCT15/WCT30 for IBUC debut and all included WC milestones, with younger ages for the best performance group. These results demonstrate that even if the junior WCH results showed a low correlation with future WCT performance, the early twenties seem to be an important age to get the first chance to compete in the WC. One explanation could be that an athlete may need to develop an understanding of the (competition) level for the WC. This facilitates a drive to train and to develop physiologically and mentally for future success. This is partly supported by the *Goal setting theory* by Locke and Latham (25, 26). Important parts of the *Goal setting theory* are, e.g., that the goals are specific, challenging, and to use short-term goals in addition to the long-term goals. According to Locke and Latham (25) it is also important to provide timely feedback showing performance or progress toward the goal. This has also been shown to be an effective strategy in achieving the desired goal in previous intervention studies [see review by Jeong et al. (27)]. One way to do this is by using a feedback chart, which plots performance over time to reveal three things: (1) how well the person is performing now, (2) how far away the goal is, and (3) whether the person is making progress towards the goal (25). If a biathlete is getting the chance to compete in the WC at a young age, these three points may be easier to state which might be helpful in developing strategies for future performance by creating an understanding of their own capacity in relation to the desired goal. The age at peak performance seems to be relatively high in distance XC skiing (∼26 years) (28), while two of the best biathletes of all time had their peak performance at ages 24–30 years (4) and 30–33 years (5), respectively. However, to reach success in the late twenties/early thirties, the training and physiological development in the late adolescent and early twenties may be of great importance.

The skiing speed is an important part of biathlon performance, explaining ∼60% of the variation in sprint performance between biathletes who placed top 10 and 20–30 in World Cup competitions (29). The importance of shooting accuracy increases in other competition formats (individual pursuit and mass-start), explaining ∼50% of the variation in overall performance (30–32). Earlier studies on XC skiing have shown that both the aerobic capacity and the laboratory-based skiing performance increase from late adolescence (junior) to mid/late 20s (senior) (33–36). Rusko (33) also demonstrated that XC skiers at an international senior level were able to increase their maximal oxygen uptake at higher ages compared to their less successful counterparts. Similar patterns have been shown in other sports, where successful senior athletes specialize in the main sport later and continuously improve their performance also at older ages (9, 21, 22, 24, 37). Successful senior athletes have also been shown to be able to increase their training volume more during late adolescence compared to their near-elite counterparts (23). Research in biathlon and XC skiing has shown that athletes progressively increase their annual endurance training volume with 20–50 h·year^−1^ during late adolescent/early twenties (5, 38, 39). Some studies have also demonstrated further increases in training volume of 20–40 h·year^−1^ up to ages of 27–35 years (4, 5, 36, 40). An increase in training volume during late adolescence seems to be crucial for future success, and Kårström et al. (41) showed that biathletes who reached the national team in Sweden, had a higher training volume during the last year of upper secondary school (age 19–20 years) compared to their less successful counterparts, with no difference in training volume during the earlier school years. A previous meta-analysis by Güllich and Barth (20) has also demonstrated that greater-performing seniors began talent promotion programs at an older age compared to high-performing juniors. Taken together, since the early 20s seem to be an important age for future biathlon success, involvement in elite support programs in biathlon may be of greatest importance in the ages ∼20 years. Some nations (like the United States of America) have a much more systematic long-term athlete development model, compared to other European countries, with a collegiate sport system to help the athletes (normally aged 18–24 years) combine high-level sport and education which has been discussed by Yustres Amores et al. (42). Such a system could be beneficial to implement in biathlon since this is a crucial time for performance progression and the age when many biathletes are done with upper secondary school, or equivalent, and need to take a higher responsibility for their own sports career.

### Practical applications

This study shows that the relation between junior and senior performance is low, but that the age of the athlete when reaching certain WC performance milestones seems to be of importance. The milestones have been reached in the early twenties for the most successful biathletes in WCT, which indicates that the development in training and performance during late adolescence seems to be of higher importance than the actual ranking in the youth and junior WCH itself. From a coach's (or coaching) perspective, it may be of great importance to progressively help the athletes to further develop the training volume and quality during adolescence and to make sure that they have the support they need also after they finish upper secondary school in the age of 19–20 years. From the perspective of a biathlon federation, it is important to support the biathletes in the early twenties (after upper secondary school) and to give opportunities for further biathlon development. Due to the results of this study, it may be wise not to choose athletes for a team or training group only based on the ranking in the youth or junior WCH, but to also take the potential training- and physiological development into consideration to create future biathlon success.

### Limitations

This study is built on a large dataset with results from the top 30 biathletes in the WCT during the last 10 years. However, there are some limitations that are worth taking into consideration. First, the results of the junior WCH might be slightly harder to interpret since some of the best biathletes are competing in the senior WCH/WC during the last years of the junior age, instead of the junior competitions. Secondly, the junior age in biathlon is relatively high (20–22 years) compared to the reference literature where the transition from junior to senior is normally at the ages of 18–20 years old. This makes the comparison between different sports harder. Thirdly, in this study, the results from WCT are used as a senior performance indicator, instead of results from the WCH or Olympic Games. The WCT ranking was used since the overall performance during the whole competition season is highly ranked in biathlon by athletes and coaches. However, the results might have turned out differently if single results from championships were used instead.

## Conclusion

A high proportion of the biathletes ranked in the top 30 in the WCT competed in the youth and/or junior WCH, but the correlation between junior and senior WCT performance was low. Taken together, participation in youth and junior WCH seems to be of higher importance than the result itself for future biathlon success. However, the biathletes ranked top 6 in the WCT reached multiple WC performance milestones (top 40, 15, 6, and 3) at a lower age compared to the lower WCT performance groups. Moreover, a younger age when reaching the top 15 in WC for the first time was associated with a higher chance of reaching the best WCT performance group in the future.

## Data Availability

The raw data supporting the conclusions of this article will be made available by the authors, without undue reservation.

## References

[1] Laaksonen MS Finkenzeller T Holmberg HC Sattlecker G. The influence of physiobiomechanical parameters, technical aspects of shooting, and psychophysiological factors on biathlon performance: a review. J Sport Health Sci. (2018) 7:394–404. 10.1016/j.jshs.2018.09.003 30450247 PMC6234024

[2] IBU. IBU Event and Competition Rules. Salzburg, Austria: International Biathlon Union (2024). Available online at: https://www.biathlonworld.com/inside-ibu/downloads (accessed 2024-05-03).

[3] Dzhilkibaeva N Ahrens M Laaksonen MS. Can performance in biathlon world cup be predicted by performance analysis of biathlon IBU cup? Int J Perform Anal Sport. (2019) 19:856–65. 10.1080/24748668.2019.1665884

[4] Schmitt L Bouthiaux S Millet GP. Eleven years’ monitoring of the world’s most successful male biathlete of the last decade. Int J Sports Physiol Perform. (2021) 16:900–5. 10.1123/ijspp.2020-0148 32887848

[5] Solli GS Flom AH Talsnes RK. Long-term development of performance, physiological, and training characteristics in a world-class female biathlete. Front Sports Act Living. (2023) 5:1197793. 10.3389/fspor.2023.1197793 37398554 PMC10308379

[6] IBU. Datacenter. Salzburg, Austria: International Biathlon Union (2024). Available online at: https://biathlonresults.com/#/start (accessed 2024-05-03).

[7] Barth M Güllich A Macnamara BN Hambrick DZ. Quantifying the extent to which junior performance predicts senior performance in Olympic sports: a systematic review and meta-analysis. Sports Med. (2023) 54(1):95–104. 10.1007/s40279-023-01906-0 37676619 PMC10799111

[8] Li P De Bosscher V Pion J Weissensteiner JR Vertonghen J. Is international junior success a reliable predictor for international senior success in elite combat sports? Eur J Sport Sci. (2018) 18:550–9. 10.1080/17461391.2018.1439104 29490566

[9] Barth M Güllich A Macnamara BN Hambrick DZ. Predictors of junior versus senior elite performance are opposite: a systematic review and meta-analysis of participation patterns. Sports Med. (2022) 52:1399–416. 10.1007/s40279-021-01625-4 35038142 PMC9124658

[10] Cohen J. Statistical Power Analysis for the Behavioural Sciences. 2nd ed. Hillsdale: Lawrence Earlbaum Associates (1988).

[11] Güllich A Barth M Macnamara BN Hambrick DZ. Quantifying the extent to which successful juniors and successful seniors are two disparate populations: a systematic review and synthesis of findings. Sports Med. (2023) 53:1201–17. 10.1007/s40279-023-01840-1 37022588 PMC10185603

[12] Brustio PR Cardinale M Lupo C Boccia G. Don’t throw the baby out with the bathwater: talent in swimming sprinting events might be hidden at early age. Int J Sports Physiol Perform. (2022) 17:1550–7. 10.1123/ijspp.2021-0530 35894878

[13] Brustio PR Cardinale M Lupo C Varalda M De Pasquale P Boccia G. Being a top swimmer during the early career is not a prerequisite for success: a study on sprinter strokes. J Sci Med Sport. (2021) 24:1272–7. 10.1016/j.jsams.2021.05.015 34099366

[14] Yustres I Del Cerro JS Psycharakis S González-Mohíno F González-Ravé JM. Swimming world championships: association between success at the junior and senior level for British swimmers. Int J Environ Res Public Health. (2021) 18:1237. 10.3390/ijerph18031237 33573125 PMC7908464

[15] Yustres I Martín R Fernández L González-Ravé JM. Swimming championship finalist positions on success in international swimming competitions. PLoS One. (2017) 12:e0187462. 10.1371/journal.pone.0187462 29108018 PMC5673220

[16] Yustres I Santos Del Cerro J Martín R González-Mohíno F Logan O. González-Ravé JM. Influence of early specialization in world-ranked swimmers and general patterns to success. PLoS One. (2019) 14:e0218601. 10.1371/journal.pone.0218601 31220159 PMC6586317

[17] Barreiros A Côté J Fonseca AM. From early to adult sport success: analysing athletes’ progression in national squads. Eur J Sport Sci. (2014) 14(Suppl 1):S178–82. 10.1080/17461391.2012.671368 24444203

[18] Yustres Amores I Santos Del Cerro J González-Mohíno F Hermosilla F González-Ravé JM. Modelling performance by continents in swimming. Front Physiol. (2023) 14:1075167. 10.3389/fphys.2023.1075167 37288433 PMC10242027

[19] Born DP Björklund G Lorentzen J Stöggl T Romann M. Specialize early and select late: performance trajectories of world-class finalists and international- and national-class swimmers. Int J Sports Physiol Perform. (2024) 19:164–72. 10.1123/ijspp.2023-0171 38061353

[20] Güllich A Barth M. Effects of early talent promotion on junior and senior performance: a systematic review and meta-analysis. Sports Med. (2023) 54(3):697–710. 10.1007/s40279-023-01957-3 37921913 PMC10978645

[21] Güllich A Macnamara BN Hambrick DZ. What makes a champion? Early multidisciplinary practice, not early specialization, predicts world-class performance. Perspect Psychol Sci. (2022) 17:6–29. 10.1177/1745691620974772 34260336

[22] Güllich A. International medallists’ and non-medallists’ developmental sport activities—a matched-pairs analysis. J Sports Sci. (2017) 35:2281–8. 10.1080/02640414.2016.1265662 27923322

[23] Moesch K Elbe AM Hauge ML Wikman JM. Late specialization: the key to success in centimeters, grams, or seconds (cgs) sports. Scand J Med Sci Sports. (2011) 21:e282–90. 10.1111/j.1600-0838.2010.01280.x 21401722

[24] Born DP Stöggl T Lorentzen J Romann M Björklund G. Predicting future stars: probability and performance corridors for elite swimmers. J Sci Med Sport. (2024) 27(2):113–8. 10.1016/j.jsams.2023.10.017 37968181

[25] Locke EA Latham GP. The application of goal setting to sports. J Sport Exerc Psychol. (1985) 7:205–22. 10.1123/jsp.7.3.205

[26] Locke EA Latham GP. Building a practically useful theory of goal setting and task motivation: A 35-year odyssey. Am Psychol. (2002) 57:705–17. 10.1037/0003-066X.57.9.705 12237980

[27] Jeong YH Healy LC Mcewan D. The application of goal setting theory to goal setting interventions in sport: a systematic review. Int Rev Sport Exerc Psychol. (2023) 16:474–99. 10.1080/1750984X.2021.1901298

[28] Walther J Mulder R Noordhof DA Haugen TA Sandbakk Ø. Peak age and relative performance progression in international cross-country skiers. Int J Sports Physiol Perform. (2022) 17:31–6. 10.1123/ijspp.2021-0065 34186511

[29] Luchsinger H Kocbach J Ettema G Sandbakk Ø. Comparison of the effects of performance level and sex on sprint performance in the biathlon world cup. Int J Sports Physiol Perform. (2018) 13:360–6. 10.1123/ijspp.2017-0112 28771061

[30] Björklund G Dzhilkibaeva N Gallagher C Laaksonen MS. The balancing act between skiing and shooting—the determinants of success in biathlon pursuit and mass start events. J Sports Sci. (2022) 40(1):96–103. 10.1080/02640414.2021.1976493 34553677

[31] Luchsinger H Kocbach J Ettema G Sandbakk Ø. The contribution from cross-country skiing and shooting variables on performance-level and sex differences in biathlon world cup individual races. Int J Sports Physiol Perform. (2019) 14:190–5. 10.1123/ijspp.2018-0134 30039989

[32] Luchsinger H Kocbach J Ettema G Sandbakk Ø. Contribution from cross-country skiing, start time and shooting components to the overall and isolated biathlon pursuit race performance. PLoS One. (2020) 15:1–12. 10.1371/journal.pone.0239057 PMC7489554 32925963

[33] Rusko H. The effect of training on aerobic power characteristics of young cross-country skiers. J Sports Sci. (1987) 5:273–86. 10.1080/02640418708729782 3453408

[34] Rusko HK. Development of aerobic power in relation to age and training in cross-country skiers. Med Sci Sports Exerc. (1992) 24:1040–7. 10.1249/00005768-199209000-00014 1406188

[35] Sollie O Losnegard T. Sex differences in physiological determinants of performance in elite adolescent, junior, and senior cross-country skiers. Int J Sports Physiol Perform. (2022) 17:1304–11. 10.1123/ijspp.2021-0366 35894954

[36] Walther J Haugen T Solli GS Tønnessen E Sandbakk Ø. From juniors to seniors: changes in training characteristics and aerobic power in 17 world-class cross-country skiers. Front Physiol. (2023) 14:1288606. 10.3389/fphys.2023.1288606 38054044 PMC10694351

[37] Güllich A Barth M Hambrick DZ Macnamara BN. Participation patterns in talent development in youth sports. Front Sports Act Living. (2023) 5:1175718. 10.3389/fspor.2023.1175718 37274619 PMC10232881

[38] Karlsson Ø Laaksonen MS Mcgawley K. Training and illness characteristics of cross-country skiers transitioning from junior to senior level. PLoS One. (2021) 16:e0250088. 10.1371/journal.pone.0250088 33989314 PMC8121355

[39] Osborne JO Solli GS Engseth TP Welde B Morseth B Noordhof DA Annual volume and distribution of physical training in Norwegian female cross-country skiers and biathletes: a comparison between sports, competition levels, and age categories-the FENDURA project. Int J Sports Physiol Perform. (2024) 19:19–27. 10.1123/ijspp.2023-0067 37917966

[40] Solli GS Tønnessen E Sandbakk Ø. The training characteristics of the world’s most successful female cross-country skier. Front Physiol. (2017) 8:1069. 10.3389/fphys.2017.01069 29326603 PMC5741652

[41] Kårström A Laaksonen MS Björklund G. School’s out for summer-differences in training characteristics between adolescent biathletes of different performance levels. PLoS One. (2023) 18:e0290408. 10.1371/journal.pone.0290408 37616200 PMC10449162

[42] Yustres Amores I Santos Del Cerro J Rodrigo Carranza V Hermosilla-Perona F. Early specialization and progress of finalist swimmers in world championships and Olympic games. J Funct Morphol Kinesiol. (2024) 9(4):187. 10.3390/jfmk9040187 39449481 PMC11503418

